# Grade IV blunt splenic injury – the role of proximal angioembolization. A case report and review of literature

**Published:** 2013-12-25

**Authors:** I Gheju, MD Venter, M Beuran, L Gulie, I Racoveanu, P Carstea, I Iftimie Nastase, DP Venter

**Affiliations:** * 3rd Department of Surgery, Clinical Emergency Hospital, Bucharest, Romania; ** “Carol Davila" University of Medicine and Pharmacy, Bucharest, Romania; *** 1st Department of Interventional Radiology, Clinical Emergency Hospital, Bucharest, Romania; **** Department of Radiology, Clinical Emergency Hospital, Bucharest, Romania

**Keywords:** severe splenic laceration, non-operative management, angio-embolization

## Abstract

Abstract

The authors present a case of grade IV traumatic spleen rupture (AAST-OIS) and an Injury Severity Score of 21 and a Revised Trauma Score RTS=7.841, which was managed without surgery, but with proximal splenic angioembolization (SAE), with a positive outcome. Indications, types and side-effects of SAE are also discussed with regard to blunt spleen trauma and the benefits of SAE as non-operative treatment approach. It is the first case of a grade IV splenic laceration non-operatively managed to be published in Romania.

The treatment of blunt splenic trauma has significantly changed over the last decades, when the non-operative management approach was first employed. SAE, as part of the NOM protocol, has enabled surgeons to treat many patients with severe blunt splenic injuries (grade III, IV) who are hemodynamically stable or easy to stabilize in a conservative manner. Thanks to SAE, there has been an increase in the number of patients treated non-operatively and a decrease of therapy-associated failures (due to undiagnosed splenic vascular injuries that progressed). 

 Non-operative management of blunt splenic trauma is the safest method to preserve the organ itself and to prevent the unwanted consequences which occur after spleen removal.

## Case presentation

 A 27-year-old male was admitted in the Department of Surgery (Clinical Emergency Hospital, Bucharest) after a fall from height (8 meters) with multiple trauma, haemoperitoneum after splenic rupture, blunt bladder injury with microscopic hematuria, mild cerebral contusion (retrograde amnesia), facial abrasions, blunt chest trauma and blunt right knee injury.

Past medical history included hepatitis C virus infection.

 On admission: GCS=15, retrograde amnesia, hemodynamically stable (BP=122/70 mmHg; HR=75/min, sinus rhythm; RR=14/min), abdomen soft, no rebound or guarding, tender in the left upper quadrant and epigastrium. The abdominal ultrasound showed perisplenic fluid (3/15 mm) and a hypoechoic splenic area of 2/12 mm.

**Fig. 1:**
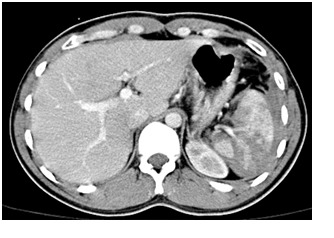
Prevalence of CMBs in patients with small vessels disease

**Fig. 2:**
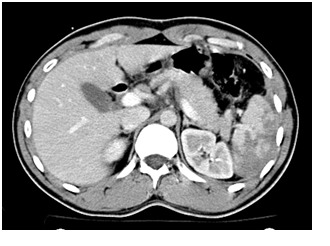
ICH volume (cm3) in patients with and without CMBs

**Fig. 3:**
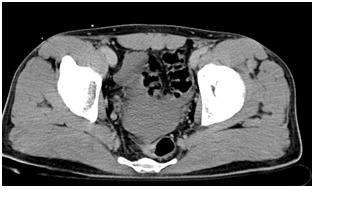
Average volume of ICH (cm3) according to location

Contrast CT showed extensive splenic laceration exceeding 60% of the spleen, sparing the upper pole, with capsule disruption but sparing the splenic pedicle, perihepatic and perisplenic haemoperitoneum (**Fig. 1,Fig. 2**); blood in the pelvis (**Fig. 3**).

**Fig. 4:**
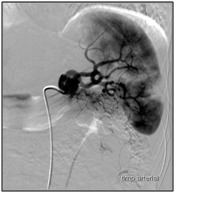
Average volume of ICH (cm3) according to location and presence of CMBs

The presence of a grade IV splenic injury (AAST-OIS) with a moderate haemoperitoneum imposed the performance of splenic angiography that revealed a heterogeneous contrast uptake within the splenic parenchyma with fine areas of contrast extravasation (**Fig. 4**). A proximal SAE was performed by using a fibrin sealant TachoSil ®; a final angiographic check did not highlight any other areas of contrast extravasation.

 The post-procedure progress was very good. A repeat CT exam on day 9 showed a normal post-embolization aspect. A peripheral blood smear taken on day 10 post-embolization did not show any Howell-Jolly antibodies.

## Discussion

According to Lucas [**1**], the initiator of non-operative management of splenic trauma was Wanborough, in 1940 (Sick Children's Hospital Toronto).

In 1968, Upadhyaya said that " ... very often, in children with splenic trauma, a significant blood loss is not apparent. It is interesting that the majority of children in this series had no splenic bleeding at the time of laparotomy " [**2**]. This is explained by various mechanisms: hypotension, clots, regional blocking by the greater omentum, containment and local pressure effect produced by the newly developed perisplenic hematoma and the presence of an intact splenic capsule.

 In 1971, Douglas and Simpson from the Toronto Hospital for Sick Children, described 32 children with clinical signs of splenic injury, who were non-operatively treated; 25 of them did not require surgery. This study proved that an injured spleen can heal spontaneously and in most cases, the recovery being uneventful [**3**].

In traumatic spleen injuries in adults, the surgeons were initially reluctant in selecting the non-operative treatment for the following reasons: the post-splenectomy sepsis was less common and less dangerous than in children; age-related architectural and vascular changes within the spleen are less likely to produce a spontaneous hemostasis; the risk of omitting associated lesions; the possibility of a DRS (delayed rupture of the spleen) and the occurrence of posttraumatic pseudocyst or splenosis [**4**].

 Patients with traumatic splenic injuries can be treated surgically or non-operatively, according to the patient’s, surgeon’s or hospital’s characteristics. Britt [**5**] introduced the term “alternative surgery" in order to define the non-operative treatment or the selective approach of trauma patients.

 NOM is the updated concept of SOS-“Save Our spleens “ - (originally applied to children) and one which, some surgeons have had the courage to apply to adults.

 The situation has changed after Sclafani performed a successful proximal splenic angioembolization in adult patients with spleen trauma [**6-8**]. The method was subsequently used in children but with a much lower frequency and indications yet unclear.

 Currently, it is considered that angiography (both diagnostic and therapeutic) is part of splenic NOM algorhythm for spleen trauma [**9-11**]. The method is indicated in hemodynamically stable patients or easy to stabilize. Hypotension upon admission is a fiercely debated issue particularly because it may be caused by various factors. Hagiwara [**12**] and Bee [**13**] showed that hypotension (as a single parameter) is not a prognostic factor for the failure of NOM. Similarly, Chen [**14**] stressed that hypotension, if correctly interpreted and treated, is not a contraindication for SAE.

 Indications for splenic angiography are the following [15-18]:

 - splenic injury grades 3, 4, 5;

 - vascular lesions seen on the initial CT, regardless of severity;

 -active bleeding obvious on CT or “contrast blush" in hemodynamically stable patients;

 - an unexplained Hct decline in the absence of other injuries;

 - severe haemoperitoneum.

 SAEs increased the NOM’s success by stopping the bleeding and preventing the delayed rupture of the spleen [**19**].

 Currently, various materials have been used for splenic embolization, such as metallic coils, fragments of hemostatic agents (“Gelfoam pledgets", TachoSil ®) with a diameter of more than 1,000 μm, which injected through the catheter occlude the vessel achieving hemostasis (special MRI-compatible coils were created which would allow further MRI examinations) or microspheres. The main advantage of these hemostatic agents is that after a few weeks, they are reabsorbed through the action of macrophages, thus achieving repermeabilization of the blood vessel [**20**]. However, due to this exact characteristic, some authors [**20-22**] actually contraindicate these hemostatic agents (increased rate of re-bleeding). Similarly, Smith [**10**] reported excellent results with the use of metal coils. Haan [**23**] reported an increased frequency of splenic infarction after using Gelfoam.

 Finding traumatic intra-splenic vascular lesions is still an important topic of debate.

 Omert et al [**23**] considered the “contrast blush" a common finding in severe splenic injuries, yet it does not represent a surgical indication per se. Contrast blush is defined as a contrast focusing within the parenchyma or extra-capsular found on early or late CT films.

 Burlew et al [**24**] found that intrasplenic contrast extravasation is not an absolute indication for SAE / surgery. The study was based on the finding that not all trauma patients transferred from Denver Health Medical Center for spleen angioembolization had contrast blush on repeat CT examination. Therefore, not all contrast-blushes are equivalent, some resolving spontaneously through intra-parenchymal tamponade effect. This result should, however, be regarded with some reservation due to the retrospective character of the study, the small number of cases and the lack of specification regarding the CT exam parameters (the amount of contrast administered, infusion rates, section thickness, late images).

 The major problem with this algorhythm is patient selection criteria (repeat CT exam?, Doppler Ultrasound?, Ultrasound with contrast?).

 Omert [**23**] states that the patient's hemodynamic parameters have a higher predictability than the isolated presence of contrast-blush to establish therapeutic indications; the mere presence of contrast-blush is not an absolute indication for SAEs. A similar result has been reported in pediatric traumatic spleen pathology [**25**].

 The authors’ recommendations in these cases are: repeat, careful, clinical and laboratory observations, dynamic FAST (especially in cases with initial negative FAST ultrasound results) and, in some cases, abdominal CT exam.

 Recently Michailidou and Velmahos [**26**] found in a retrospective study that not all contrast extravasationes (as shown at MDCT-64 multidetector scanner) require interventional radiology / surgery. These high-speed scanners can identify very small contrast extravasations and with current unclear significance. The authors evaluated IV contrast according to location (organ, pelvis and other locations), to size (small <1.5 cm large ≥ 1.5 cm), free peritoneal fluid versus intraparenchymal blush and according to number (single, multiple); a late blush was considered at 3 hours after admission. Finally, 43.5% of the cases did not require any therapeutic intervention. The statistical analysis revealed three independent factors that were highly predictive for therapeutic intervention: SBP ≤ 100 mmHg upon admission, large blush and abdominal AIS ≥ 3; simultaneous presence of all 3 was associated with therapeutic intervention in 100% of the cases whilst their absence was associated with an intervention in 31% of cases. A very interesting finding was that 73% of the splenic injuries with contrast blush required therapeutic interventions and of these, 50% had small blushes (<1.5 cm) and 82% had large blushes (≥ 1.5 cm).

 Bhullar [**27**] noticed that out of 1056 patients with blunt spleen trauma, 95 (17%) had contrast blush on CT examination and in 97.7% blood extravasation was confirmed on angiography. 9.5% of the patients treated non-operatively had severe lesions (grades IV-V) without contrast blush on CT out of which, 20 (85%) had angiography-proven active bleeding. For these patients, SAE has been successful, however, in 26% of those cases with severe injuries and without contrast blush and without splenic angiography, NOM was a failure. The author concluded that the SAE is required in all splenic contusions with contrast blush, regardless of the lesion severity. Also, in severe splenic injuries (grades IV-V) managed with NOM, SAE is required, even without contrast blush on CT scan.

The final conclusion belongs to Scale (Michailidou-discussion 26): as not all organs are identical, current MDCT shows vascular lesions that would not have been observed in the past, but whose therapeutic value were questionable / minor. Thus, we can only agree with Marmery [**29**], who proposed a new CT classification of traumatic spleen injuries, which would include active bleeding, arterio-venous intrasplenic fistula, pseudoaneurysm and vascular lesions as essential elements for the correct assessment of injury extent.

 Willman (cit. 26) reports that “when IV contrast extravasation is found, surgery /immediate therapeutic angiography is needed. Fang [**28**] reports that in the presence of IV contrast extravasation “non-operative treatment should be stopped and emergency angiography / surgery should be performed without delay." Have these findings become history? Michailidou [**26**] showed that most of these findings are based on large blushes (≥ 1.5 cm), which were, in fact, the only ones detected with CT scanners at that time and which would, obviously, suggest an active bleeding. These findings are no longer accurate in the era of high-speed MDCT, which can highlight minimal bleeding with little/ no consequences. Thus, the authors’ conclusion is similar to Omert’s [**23**]: “the presence of contrast blush is not an absolute indication for surgery / angiography".

 Clinically, the SAE should be performed as soon as the contrast extravasation is found (abdominal CT examination) and before the onset of hemodynamic instability [**17**].

 According to Schurr's [**30**] and Bhullar [**27**] the presence of contrast blush increases the risk of NOM failure by 22 to 24 fold. 

 SAE may be carried out:

 • in an emergency (“acute")-in hemodynamically stable patients with active bleeding or severe splenic injuries (III-V);

 • delayed (“subacute")-indications are represented by declining of Hb values during NOM or finding a pseudoaneurysm (> 1.5 cm) or the occurrence of a contrast blush on repeat CT examination.

 Splenic angio-embolization (SAEs) can be carried out:

 • distally (supra-selective)-achieves containment of damaged vessel, retains a normal blood flow to an important splenic area but is a time-consuming procedure and requires high technical skills [**31**].

 • Proximally (the trunk of splenic artery, distal to the dorsal pancreatic artery) is performed with metal coils or absorbable hemostatic materials (Gelfoam-Pharmacia, Kalamazoo,MI; Tacho-comb) and achieves hemostasis by decreasing arterial blood flow and intra-splenic pressure, which promotes clot formation and wound healing. The spleen viability is ensured by collateral circulation (branches of the left gastric artery, gastro-epiploic arteries, omental, pancreatic, short gastric arteries) as shown by experimental animal studies (12, Anderson cit. 32). In human studies, it was found that there was a decline of intrasplenic pressure by 47-58% (Bessoud cit. Zmora 32). Sclafani [**8**] believes that this procedure is compatible with the maintenance of splenic immune function and even if surgery is necessary, splenorrhaphy is facilitated. Proximal SAE is faster, easier to perform and is associated with a lower NOT-related failure rate and a reduced incidence of post-procedural complications (abscess, splenic infarction) [10,19,22,23,33]. According to Van der Vlies [**19**], the only drawback of proximal SAE is that, in the event of a possible re-bleeding, supraselective embolization is difficult / impossible to perform.

 The lack of highlighting of left gastric artery or its tributaries on angiography may be a cause of proximal post -SAE splenic infarction [**32**]. This can be explained by compromised collateral circulation secondary to age-related splenic pathology. It should be noted that the presence of a patent left gastric artery may be of particular importance to maintaini collateral circulation after proximal splenic SAE [**32**]. For this reason, it is indicated that a celiac trunk arteriography is obtained with special focus on the left gastric artery [**34**].

Splenic artery occlusion was originally used to control hypersplenism associated with liver cirrhosis and portal hypertension syndrome. Hypersplenism recurrence suggests the development of collateral circulation, which in Harbrecht’s opinion [**31**], would allow for the traumatized spleen to keep its functions.

 • Combined

 Angiographic examination is performed after the CT scan revealed intra-splenic vascular lesions in order to confirm and potentially embolize them at the same time. Embolization is performed only on the basis of angiographic lesion diagnosis [15,34].

 After embolization, patients were admitted to ICU, where their vitals, blood count (dynamic values of hemoglobin) and clinical status (repeat abdominal examinations) were closely monitored.

 There are views (Sclafani-8) who argue that proximal SAE causes fewer complications and increases the NOM’s success rate (by decreasing blood flow and intrasplenic pressure -47-58% - facilitates clot formation and the collateral circulation ensures viability of the spleen). SAE may cause distal necrosis / infection, in keeping with the avascular area. A tortuous splenic artery makes distal angioembolization difficult / impossible, thus being an indication for proximal SAE only.

 Repeated embolization (“second-look" angiography) is indicated in recurrent bleeding and after an initial negative angiography (10%) [**35**]. Haan is partial to distal embolization for mild and combined splenic injuries (with no significant statistical differences). According to the same author [**35**] “delayed vascular emergencies" (term introduced by the Memphis group) are actually diagnostic delays which may be shown by angiography in severe splenic lesions (grade 3,4,5). The Memphis Group (Davis, Fabian, Croce) showed that there may be vascular lesions, initially not identified on CT or angiographic exams due to arterial spasm at the time of the examination, and subsequently become symptomatic. Repeated spiral CT identified 80% of the initially missed vascular lesions (used as a screening test for angiography). The only statistically significant marker of NOM failure is the arterio-venous fistula for which the proximal embolization is not sufficient, requiring a more direct approach-the distal embolization [**36**].

 The findings of Haan [**37**] are:

 - proximal embolization (decreases splenic perfusion pressure) is a therapeutic modality much more useful than distal embolization, except for the AV fistula;

 - the immunological consequences of proximal embolization are unclear and require further study;

 - the use of SAE decreases by 20% the failure rate of NOM in grades 4 and 5 splenic injuries;

 - the SAE would be superior to surgery in the treatment of blunt splenic injuries in multiple trauma patients with head injuries.

 - SAE is a useful and effective method as part of the NOM protocol but employed in just 7% of cases.

 Haan [**22**] abandoned selective SAE in favor of proximal SAE, performed with metal coils, however finding a new category of patients: with persistent post-procedural pseudoaneurysm (PSA) or new postprocedural PSA. In these patients, the success rate of NOM was 91% and the spleen preservation was achieved in 94% of cases.

 The indications of SAE are [**20-22,36-38**]:

 • proximal SAE: 

 o hilar lesions;

 o >3 separate peripheral vascular lesions;

 o An injury affecting> 50% of spleen;

 o an AV fistula, PSA;

 o a vascular injury with angiographic appearance of amputation (suggestive lesion associating vascular spasm);

 o a technical impossibility of performing distal SAE.

 • Selective SAE: limited splenic vascular lesions;

 Benefits: ensures hemostasis and normal perfusion of the remaining organ.

 • combined SAE: multiple vascular lesions (severe injury degrees), intraperitoneal extravasation.

 After SAE, repeat CT (postprocedure) focuses on finding: persistent vascular injury, pseudoaneurysm formation, infracted area size, evidence of local infection (splenic abscess).

 Hagiwara [**12**] recommends a repeated CT scan (in patients with SAE) between days 10 and 15 post-SAE in order to assess perfusion and splenic blood flow through collateral circulation. The splenic reticuloendothelial function was investigated by scintigraphy with colloid sulfur Tc-99m having been performed between days 10 and 15 as well. Currently, the updated indications for NOM include diagnostic / therapeutic angiography in carefully selected unstable trauma patients [**12-39**]. 

 A study by Wei showed that [**11**]:

 • There were no patients with transient hypotension in which SAE failed - 31% of patients who underwent SAE with good results were over 55 years old;

 • The spleen injury score was higher (3.8) than that of patients undergoing emergency surgery (3.4);

 • in 68% of the cases a distal SAE was performed with an average of 50% embolized splenic area;

 • reduced packed red cell / first 24 hours transfusion needs than operated patients;

 • radiological costs were similar to the surgical ones; 

 • there were no differences concerning thrombo-embolic, pleural complications, mechanical ventilation-associated pneumonia and mortality rates between the 2 groups.

 The final decision depends on the experience of the trauma team involved and the technical possibilities of the trauma centers.

 Jeremitsky [**40**] believes that SAE should be indicated in cases with active bleeding or in the presence of intrasplenic pseudoaneurysm (identified on CT scan), while splenic injury extent and the degree of hemoperitoneum are not absolute indications “per se". Wei [**11**] considers that SAE is appropriate in severe injuries as well (grades IV or V) which associate severe haemoperitoneum (indication pointed out by Thompson as well- [**41**]).

 Imbrogno [**34**] believes that AES is indicated in hemodynamically stable patients in the following situations:

 - active extravasation of contrast;

 - severe splenic lesions (grade ≥ 3) and decreased hematocrit;

 - splenic vascular lesions associated with decreased hematocrit values.

 Changes in patients with normal indication of tomographic angiography but SAE are unclear.

 The most recent study on this topic [**27**] provided the following information:

 - contrast blush on the initial CT examination;

 - grade IV-V splenic lesion on the initial CT examination;

- decrease in Hb after admission, during NOM.

 The authors point out that, often, the indication of SAE involves the simultaneous presence of several factors mentioned in this paper. Their recommendation is conducting the angioembolization in all cases of splenic rupture, grades IV or V, regardless of the presence / absence of other findings and using it selectively for grade I-III injuries only if there is a contrast blush or declining Hb during NOM.

 The use of an aggressive SAE resulted in favorable NOM results in 80% of severe splenic injuries (grade IV-V) [**15**].

 Howell's study [**42**] demonstrated that a delay in interventional radiology procedure initiation in hypotensive trauma patients with an average ISS of 17 is associated with a two-fold increase in risk of death. For each hour of delay the risk of death increases with 47%, regardless of the mechanism of injury. The faster the interventional radiology procedure is performed the better the results will be.

 The technical failure of SAE is defined as the inability to cannulate and embolize the splenic artery and its branches; the SAE is deemed as failed when subsequent surgery is needed to acquire hemostasis [**18**]. Thus, SAE failure is placed by this author around 5-6%.

 Selectively implementing SAE as non-operative management for splenic trauma cases in patients at risk decreased the failure of this method to values of 2-4% [**7-8,14-15**].

 Currently, the use of SAE resulted in a decreased number of splenic operations [**19**], the frequency of emergency interventions performed have decreased from 33.3% to 11.9% after the introduction of this method [**43**].

 The disadvantages of this method are: difficult monitoring and resuscitation (in Angiography suite), the need for an interventional radiologist, time-consuming procedure (bearing the risk of a possible decompensation of the patient).

 Currently, there is a constant concern for the splenic functional changes post SAE. It is necessary to demonstrate that after angiographic maneuver, the residual splenic tissue is able to provide at least part of the previous functionality. In the absence of a normal immunocompetent spleen the risk of fulminant sepsis (OPSI) is permanent even if its frequency is below 1% (in multiple trauma adult patients).

 Harbrecht [**31**] concluded that the splenic immune function after SAE depends of the actual technique, blood vessel size and long-term development of collateral circulation. Similarly, Hagiwara [**12**] stresses the preservation of the splenic reticuloendothelial system’s function post-embolization.

 Walusimbi [**44**] studied the complete blood count and serum complement values and found no difference between patients with blunt spleen injuries that underwent SAE and those with blunt abdominal trauma without splenic involvement.

Malhotra [**45**] concluded that after SAE for blunt splenic injuries, the immune function is partially or totally preserved.

 Nakae’s [**46**] retrospective study, however, proves that the splenic preservation (through embolization, splenorrhaphy, partial splenectomy) opposed to complete splenectomy does not provide significant advantages in terms of immune function, including IgM and 14 serotypes of anti-S. Pneumoniae antibodies. The authors strongly suggest implementing preventive measures and thorough follow-up.

Post-embolization splenic immuno-competency remains questionable although there are studies that support its viability.

 However, current studies do not consider vaccination necessary after performing SAE for splenic trauma [**47-48**].

 SAE is an elegant solution for non-operative management of splenic trauma in all trauma centers.
